# Whole-genome duplication in an algal symbiont bolsters coral heat tolerance

**DOI:** 10.1126/sciadv.adn2218

**Published:** 2024-07-19

**Authors:** Katherine E. Dougan, Anthony J. Bellantuono, Tim Kahlke, Raffaela M. Abbriano, Yibi Chen, Sarah Shah, Camila Granados-Cifuentes, Madeleine J. H. van Oppen, Debashish Bhattacharya, David J. Suggett, Mauricio Rodriguez-Lanetty, Cheong Xin Chan

**Affiliations:** ^1^ School of Chemistry and Molecular Biosciences, Australian Centre for Ecogenomics, The University of Queensland, Brisbane, QLD 4072, Australia.; ^2^ Department of Biological Sciences, Biomolecular Science Institute, Florida International University, Miami, FL 33099, USA.; ^3^ Climate Change Cluster, University of Technology Sydney, Sydney, NSW 2007, Australia.; ^4^ School of Biosciences, The University of Melbourne, Parkville, VIC 3010, Australia.; ^5^ Australian Institute of Marine Science, Townsville, QLD 4810, Australia.; ^6^ Department of Biochemistry and Microbiology, Rutgers University, New Brunswick, NJ 08901, USA.; ^7^ KAUST Reefscape Restoration Initiative (KRRI) and Red Sea Research Center (RSRC), King Abdullah University of Science and Technology, Thuwal 23955, Saudi Arabia.

## Abstract

The algal endosymbiont *Durusdinium trenchii* enhances the resilience of coral reefs under thermal stress. *D. trenchii* can live freely or in endosymbiosis, and the analysis of genetic markers suggests that this species has undergone whole-genome duplication (WGD). However, the evolutionary mechanisms that underpin the thermotolerance of this species are largely unknown. Here, we present genome assemblies for two *D. trenchii* isolates, confirm WGD in these taxa, and examine how selection has shaped the duplicated genome regions using gene expression data. We assess how the free-living versus endosymbiotic lifestyles have contributed to the retention and divergence of duplicated genes, and how these processes have enhanced the thermotolerance of *D. trenchii*. Our combined results suggest that lifestyle is the driver of post-WGD evolution in *D. trenchii*, with the free-living phase being the most important, followed by endosymbiosis. Adaptations to both lifestyles likely enabled *D. trenchii* to provide enhanced thermal stress protection to the host coral.

## Teaser

Genomes of a heat-tolerant algal species revealed how whole-genome duplication has enhanced its efficiency as a coral symbiont.

## INTRODUCTION

Uncovering the foundations of biotic interactions, particularly symbiosis, remains a central goal for research, given that virtually no organism lives in isolation. Coral reefs are marine biodiversity hotspots that are founded upon symbioses involving dinoflagellate algae in the family Symbiodiniaceae (*1*). These symbionts are the “solar power plants” of reefs, providing photosynthetically fixed carbon and other metabolites to the coral host (*2*, *3*). Breakdown of the coral-dinoflagellate symbiosis (i.e., coral bleaching), often due to ocean warming, puts corals at risk of starvation, disease, and eventual death. Symbiodiniaceae microalgae are diverse, with at least 15 clades including 11 named genera (*1*, *4*–*6*), encompassing a broad spectrum of symbiotic associations and host specificity. Most of these taxa are facultative symbionts (i.e., they can live freely or in symbiosis), although exclusively symbiotic or free-living species also exist (*1*). Genomes of Symbiodiniaceae are believed to reflect the diversification and specialization of these taxa to inhabit distinct ecological niches (*7*, *8*). The genomes of symbionts, due to spatial confinement, are predicted to undergo structural rearrangements, streamlining, and rapid genetic drift (e.g., pseudogenization) (*7*). These traits are present in symbiotic Symbiodiniaceae (*8*).

Whole-genome duplication (WGD) is an evolutionary mechanism that generates functional novelty and genomic innovation (*9*, *10*) and can occur due to errors in meiosis, i.e., via autopolyploidy. Following WGD, the evolutionary trajectory of duplicated sequence regions generally proceeds from large-scale purging, temporary retention and/or divergence, to fixation (*11*). WGD-derived duplicated genes [i.e., ohnologs (*12*, *13*)] that are retained can provide a selective advantage and enhance fitness through increased gene dosage, specialization in function, and/or the acquisition of novel functions (*11*).

**Fig. 1.**
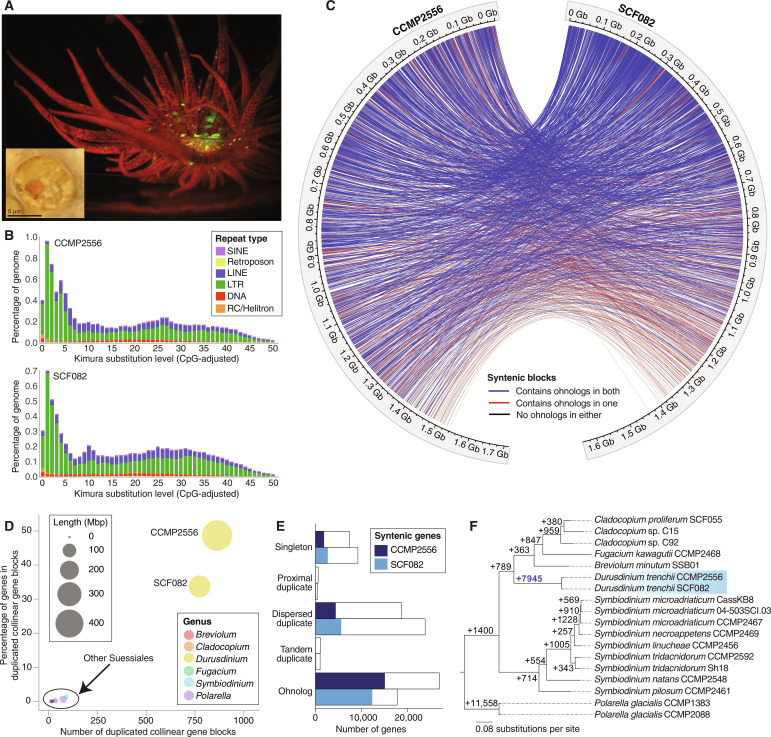
WGD in a facultative coral endosymbiont. (**A**) Microscopic images of a free-living *D. trenchii* cell and an *Exaiptasia pallida* anemone hosting *D. trenchii* under fluorescence, with red indicating the presence of *D. trenchii*. (**B**) Repeat landscapes shown separately for the CCMP2556 and SCF082 genomes. (**C**) Circle plot depicting the location of syntenic blocks containing collinear gene blocks (i.e., ohnologs) between the CCMP2556 and SCF082 genomes. Ribbons indicate syntenic gene blocks identified with MCScanX that overlap with putative WGD-duplicated regions in both isolates (blue; *n* = 2427), one isolate only (red; *n* = 612), or neither isolate (black; *n* = 35). (**D**) The percentage of genes in duplicated collinear gene blocks relative to the number of duplicated collinear gene blocks identified within the genomes of Suessiales species. (**E**) Number of genes and syntenic genes recovered for each gene duplication category for the two isolates. (**F**) Phylogenetic tree of order Suessiales showing the number of lineage-specific gene family duplications at each node.

WGD has been described in free-living unicellular eukaryotes such as yeast (*14*–*16*), ciliates (*17*, *18*), and diatoms (*19*, *20*), but not in symbiotic species. Evidence of WGD is absent in the Symbiodiniaceae, except for the genus *Durusdinium*, as observed in microsatellite sequence data (*21*–*23*). This genus includes the thermotolerant species *Durusdinium trenchii* (Fig. 1A), a facultative symbiont that confers heat tolerance to corals, thereby enhancing holobiont resilience under thermal stress (*24*, *25*). We hypothesize that WGD played a critical role in enhancing the capacity of this symbiont to confer heat tolerance to host species. Specifically, the facultative lifestyle (i.e., free-living or symbiotic) of *D. trenchii* favored fixation of WGD both during the free-living phase as an adaptation to fluctuating environmental conditions, and the symbiotic phase with an expanded gene inventory being further modified by the coral or other host species (*26*). Here, we generated de novo genome assemblies from two isolates of *D. trenchii* and analyzed their evolutionary trajectories. On the basis of gene expression profiles, we elucidate how the facultative lifestyle has contributed to the fate of ohnologs in these microalgae, and how natural selection acting on gene families has increased thermotolerance of corals hosting *D. trenchii* symbionts. These data provide strong evidence for the dual lifestyle hypothesis as a driver of post-WGD genome evolution.

## RESULTS

### Whole-genome duplication in a coral endosymbiont

We generated de novo genome assemblies from *D. trenchii* CCMP2556 (total length = 1.71 Gb; N50 = 774.26 kb) and *D. trenchii* SCF082 (total length = 1.64 Gb; N50 = 398.48 kb) using 10x Genomics linked reads (tables S1 and S2). The two genomes are highly similar in terms of whole-genome sequence (~99.7% shared identity, comparable to genomes of multiple isolates of single Symbiodiniaceae species; table S3) (*8*, *27*, *28*), size (table S4), and repeat landscapes (Fig. 1B and fig. S1), yielding ~54,000 protein-coding genes (table S5) with a comparable level of data completeness to other genome assemblies of Symbiodiniaceae (table S6; see Materials and Methods). To assess WGD in *D. trenchii*, we followed González-Pech *et al*. (*8*) to identify collinear gene blocks within each genome (Fig. 1C); these blocks likely arose via segmental duplication and/or WGD. We identified 864 blocks implicating 27,597 (49.46% of the total 55,799) genes in CCMP2556, and 776 blocks implicating 18,209 (34.02% of the total 53,519) genes in SCF082 (tables S7 and S8). The proportion of genes present in collinear blocks in *D. trenchii* is ~49-fold greater (Fig. 1D) than that in other Symbiodiniaceae and the outgroup dinoflagellate *Polarella*; these taxa have not experienced WGD, as also observed in an earlier study incorporating genomes of the free-living Symbiodiniaceae lineage, *Effrenium voratum* (*28*). We also observed a high extent of conserved synteny in *D. trenchii* (22,041 CCMP2556 genes syntenic with 21,094 SCF082 genes), with ohnologs predominant in these syntenic blocks [CCMP2556: 15,395 (69.85%C); SCF082: 12,617 (59.31%)] (Fig. 1E, fig. S2, and table S9). Using homologous protein sets derived from available whole-genome data, our inference of lineage-specific duplicated genes revealed 7945 gene duplication events specific to *D. trenchii*, which is greater than those in any other Symbiodiniaceae lineages on the tree (Fig. 1F).

Examination of the overall distribution of DNA synonymous substitutions (*K*_s_) showed a distinct peak (fig. S3), as expected following WGD; the small peak values are explained by the recency of this event in *D. trenchii* (*29*). The WGD likely occurred after the split of *D. trenchii* from its sister *Durusdinium glynnii* 0.11 million to 1.93 million years ago, based on large subunit ribosomal RNA genetic divergence estimates (*1*). Our analysis of whole-genome data following Ladner *et al*. (*30*) aligns with these estimates of a Pleistocene origin in the Indo-Pacific (see Supplementary Text), a period of frequent sea-level changes in this region (*31*). These results, based on independently assembled genomes from two isolates, combined with the extent and size of the gene blocks (table S7 and fig. S2), provide unambiguous evidence for WGD in *D. trenchii*.

### Asymmetric divergence of ohnolog-pair expression

**Fig. 2.**
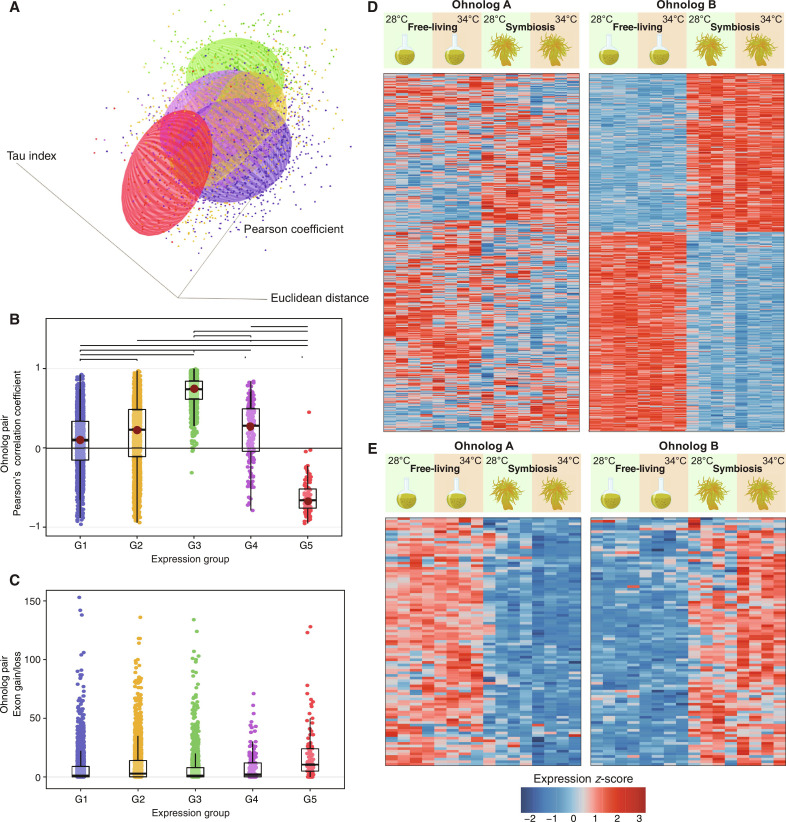
Ohnolog expression post-WGD. (**A**) Three-dimensional scatterplot of the five groups of ohnologs pairs based on their pattern of differential expression (DE), i.e., pairs for which neither gene showed DE (group 1; blue), only one showed DE (group 2; orange), both ohnologs showed DE at the same time in the same manner (group 3; green), both ohnologs showed DE but at different times (group 4; purple), and both ohnologs showed DE at the same time but in opposing directions (group 5; red). (**B**) Pearson’s correlation coefficients showing the correlation of expression patterns between each ohnolog pair within each of the five groups. (**C**) Exon gain/loss between each ohnologs pair within each of the five groups. Heatmaps depicting the normalized gene expression (*z*-score) for (**D**) groups 2 and (**E**) 5, in which ohnologs were clustered on the basis of their shared expression pattern. The assignment of gene copies (ohnolog A versus ohnolog B) for each ohnolog pair is arbitrary, based solely on common expression patterns.

To assess putative ohnolog functions in *D. trenchii*, we analyzed transcriptome data of CCMP2556 (*32*) that were generated from free-living cells in culture and from cells in endosymbiosis with the anemone *Exaiptasia pallida*, both under ambient (28°C) and thermal stress (34°C) conditions. These data (*32*), although generated from an experiment using the anemone host of *Exaiptasia* as the model for cnidarian symbiosis with Symbiodiniaceae (*33*), provide insights into how *D. trenchii* as a successful coral symbiont responds to heat stress during free-living stage and symbiotic stage. We focused on 6147 expressed ohnolog pairs that were supported by 10 or more mapped transcripts in ≥50% of the samples and inferred gene coexpression networks (fig. S4 and table S10) using weighted gene coexpression network analysis (WGCNA) (*34*). Most [4412 (71.7%) of 6147] ohnolog pairs were recovered in different networks, indicating the prevalence of expression divergence between duplicates post-WGD. We then classified ohnolog pairs into five groups based on their differential expression (DE) patterns (Fig. 2A and figs. S5 to S9; see Materials and Methods). Each group exhibited different characteristics (table S11) relative to expression (Fig. 2, A and B), sequence similarity (fig. S10), gene structure (i.e., exon gain/loss; Fig. 2C), and/or alternative splicing (figs. S11 and S12); see Supplementary Text. Ohnolog pairs that were differentially expressed between lifestyles observed in only one copy [group 2; 2244 (36.5%); Fig. 2D], and those with opposing DE observed in any one comparison [group 5; 100 (1.6%); Fig. 2E and table S12] exhibited strongly contrasting expression profiles (most Pearson’s correlation coefficients < 0; Fig. 2B). They showed significantly elevated levels of positive selection (*K*_a_*/K*_s_ = 3.31; table S11), exon gain/loss, sequence divergence, and differential exon usage (DEU; table S13) relative to the other three groups (pairwise Wilcox rank sum tests, *P* < 0.05; see Supplementary Text); these differences were not attributed to the differing number of splice junctions per gene, and ohnologs show greater extent of alternative splicing than singletons (fig. S13 and table S14).

Divergence of expression between ohnologs within a pair can have different outcomes, including the change in expression specificity, an important mechanism for adaptation after WGD. We assessed expression specificity using the τ index (*35*) that ranges between 0 (i.e., broad expression, low specificity) to 1 (i.e., narrow expression, high specificity) for all genes that passed the WGCNA quality filtering. We identified 3508 genes of high expression specificity (τ > 0.7), of which 1893 (53.96%) were ohnologs (table S15). Compared to singletons and other duplicate types (except for proximal duplicates), the ohnologs exhibited significantly elevated τ (fig. S14, Kruskal-Wallis test; *P* < 10^−5^), indicating narrow expression profiles that are more specialized to distinct conditions. This divergence in expression was observed in group 2 pairs (fig. S6), for which the differentially expressed copy in each pair showed higher τ and variance in expression relative to its counterpart (Kruskal-Wallis test; *P* < 10^−15^). Whereas most instances of specialized expression are associated with the free-living lifestyle (table S16), this specialization reflects a response to temperatures among the dispersed duplicates (i.e., duplicates separated by >20 genes; chi-square test post hoc: *P* = 0, residuals = 6.15 at 34°C) and the ohnologs (*P* < 0.05, residuals = 3.10 at 28°C). Only ohnologs displayed a tendency toward expression specificity (τ approximates 1) in the symbiotic lifestyle at both 28°C (chi-square test post hoc: *P* < 0.01, residuals = 3.61) and 34°C (*P* < 0.01, residuals = 3.89; table S16). Although the post-WGD specialization described here relates to lifestyle, parallels are known in multicellular organisms whereby the partitioning of expression across spatiotemporal scales is often observed in different tissues, organs, or developmental stages (*11*, *36*). In post-WGD yeasts, this trend may represent the uncoupling of noise and plasticity in gene expression that enables dynamic gene-expression responses in one of the two duplicates (*37*). In *D. trenchii*, this trait may provide greater flexibility in gene expression when cells are free-living or experiencing temperature stress.

**Fig. 3.**
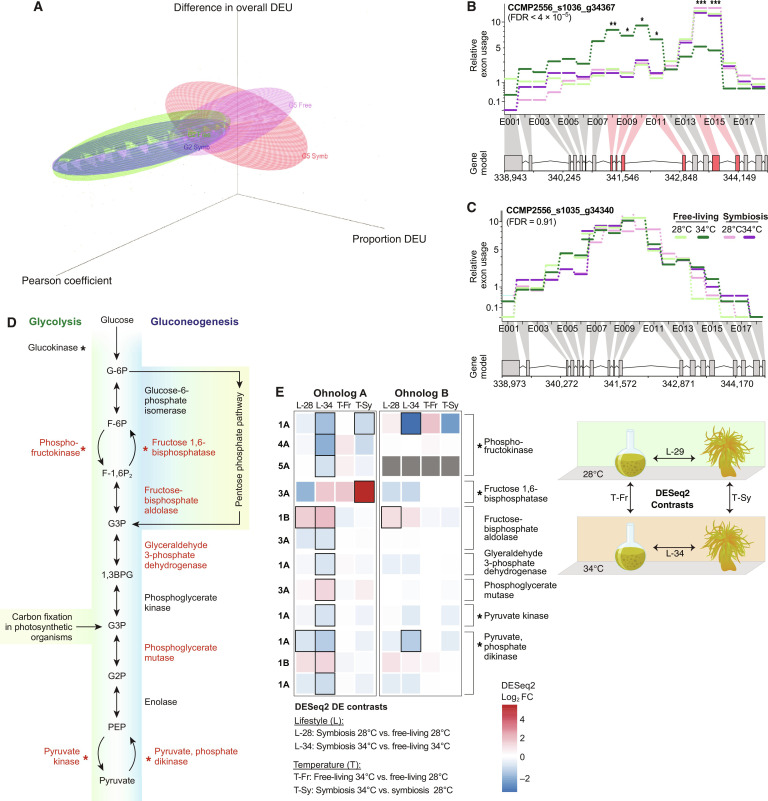
Exon reorganization underlies functional divergence. (**A**) Three-dimensional scatterplot depicting directionality of differential exon usage (DEU) among ohnologs pairs of groups 2 and 5, which reflects the pattern of gene-level DEU. The *z* axis shows the absolute change in DEU (i.e., overall DEU) within each ohnolog pair from groups 2 and 5, the *x* axis shows the relative change in DEU (i.e., the proportion of DEU), and the *y* axis indicates Pearson’s correlation coefficient of the gene expression. An ohnolog-pair encoding glutaredoxin (GRXC2) proteins from group 2 with (**B**) DEU in the ohnolog with gene-level DEU and (**C**) no DEU in its counterpart. (**D**) Glycolysis and gluconeogenesis pathways for which genes indicated in red were implicated by differentially expressed ohnologs, and an asterisk indicating rate-limiting or key enzymes. (**E**) The log_2_(fold change) in the expression of differentially expressed ohnologs across a distinct comparison of growth conditions with their corresponding scenario indicated on the right.

The up-regulation or specialization of gene expression by some ohnolog pairs to different lifestyles in *D. trenchii* appears to either be mediated by or coincide with alterations in exon organization. Evolution of WGD-derived genes can lead to loss and/or diversification of alternative spliced forms, and the partitioning of ancestral splice forms between gene duplicates. We investigated this issue by examining the interplay of sequence conservation in exonic sequences with patterns of splice junction conservation (table S17) and DEU within ohnolog pairs. On the basis of the mean percentage of shared exons per pair, the ohnolog pairs in group 5 (13.35%) had lower exonic conservation compared to other groups (>16%). DEU across all groups was biased toward functions associated with the free-living lifestyle. Nearly all ohnolog pairs in group 5 (95 of 100; Fig. 2E and fig. S9) displayed contrasting DE between free-living and symbiotic phases (e.g., a gene copy was up-regulated during the free-living phase, whereas the other was up-regulated in the symbiotic phase) at one or both temperatures. This result underscores lifestyle as a strong driver of expression divergence (see Supplementary Text). Group 5 ohnologs that are specialized for the symbiotic lifestyle exhibited lower overall DEU and had fewer exons than their counterparts that were up-regulated under the free-living lifestyle (Wilcoxon rank sum test, *P* = 0.015, *V* = 2435.5). These ohnologs also contained exons that were more dominantly expressed during the symbiotic lifestyle (Wilcoxon rank sum test, *P* = 0.02798, *V* = 2540; Fig. 3A). Such a bias in DEU composition toward symbiosis-specialized exons was not observed in the other groups, e.g., group 2 (Fig. 3, B and C). Consequently, the symbiosis-specific DEU, together with the overall decrease in per-gene exons and DEU among symbiosis-associated ohnologs in group 5, suggests a symbiosis-specific streamlining of gene function. Together with our observation of RNA editing (Supplementary Text, fig. S15, and tables S18 and S19), these results collectively indicate that alterations to gene structure and alternative splicing drive expression divergence of ohnologs in *D. trenchii* that are explained by algal lifestyle.

Although genomic streamlining is usually associated with obligate endosymbionts rather than facultative symbionts, gene duplication may facilitate streamlining in one of the two duplicates in favor of a symbiotic lifestyle. Ohnolog pairs of group 5 were significantly enriched for key functions (table S20), such as the processing of glutamine and production of the key antioxidant of glutathione, which has been linked to nitrogen cycling associated with symbiosis (*38*, *39*); the implicated genes include glutamine synthetase and *S*-formylglutathione hydrolase (table S12). These results suggest that following WGD, specialization of gene expression to distinct conditions may also be enabled by the streamlining of functions and specialization to symbiosis. In contrast, for their duplicated counterparts, the evolution of greater functional flexibility may reflect selection during the free-living phase.

### Partitioned functionality in central metabolic pathways

WGD enables the retention of complete expression networks. Of the 19 inferred coexpression networks (table S10), different gene duplication types displayed preferential distributions to WGCNA modules (*P* < 2.2 × 10^−16^, χ*^2^* = 525.63). Singletons and ohnologs were biased toward contrasting coexpression networks, with singletons predominantly associated with networks linked to the symbiotic lifestyle (M1, M8, and M17 in table S21), and ohnologs with networks linked to a free-living lifestyle (M2, M5, and M6). This result suggests that genes preferentially retained as ohnologs were expressed at contrasting times, compared to those that were lost such that the remaining copies become singletons. DE of ohnologs was observed at the greatest magnitude between lifestyles during heat stress at 34°C (chi-square test post hoc: *P* < 10^−3^, residuals = 4.03; table S22); this may explain, in part, how *D. trenchii* can establish itself or increase in abundance in new hosts both during and after heat waves (*40*–*44*). These contrasting patterns of singleton and ohnolog membership across coexpression networks indicate a strong association of ohnolog retention with expression networks that are tightly linked to the free-living lifestyle.

We investigated the retention of complete metabolic pathways in both *D. trenchii* isolates. Of the 98 pathways retained in duplicate (table S23), specialization driven by lifestyle was detected in central metabolic pathways (figs. S16 to S23) (*45*), such as glycolysis/gluconeogenesis (Fig. 3D and fig. S16). Ohnolog specialization in glycolysis/gluconeogenesis reflects the contrasting functions of this pathway during the symbiotic versus free-living phases. That is, a high rate of gluconeogenesis, inferred using ohnolog expression data, supplies glucose for translocation to the coral host during symbiosis, whereas a high rate of glycolysis fuels the energetic needs of free-living cells that tolerate more variable environments (*7*). Although most enzymes were encoded by group 2 ohnologs (for which one gene copy was differentially expressed between lifestyles; Fig. 3E), a key rate-limiting enzyme of gluconeogenesis and the Calvin cycle, fructose 1,6-bisphosphatase, was differentially expressed in response to heat stress in symbiosis. The development of minor or partitioned functionality following WGD has been described in duplicate glycolysis pathways (*46*). In yeast, these pathways diverged and became semi-independent, with each specialized for low and high glucose levels (*46*). In *D. trenchii*, this might allow fine-tuning of carbon metabolism to the contrasting energetic needs of a dual lifestyle.

## DISCUSSION

**Fig. 4.**
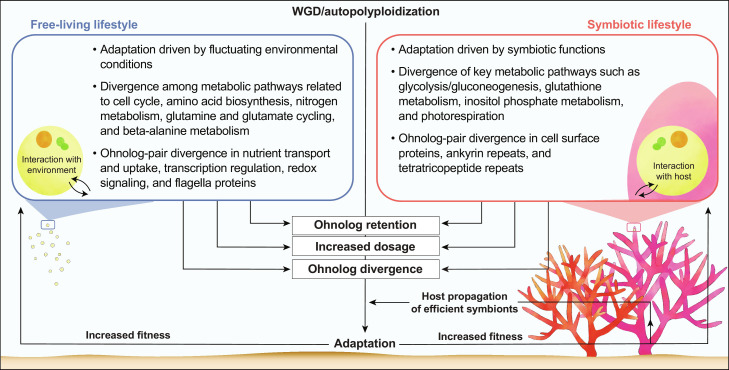
Model of divergence post-WGD in a facultative endosymbiont. Putative selective constraints faced by free-living and symbiotic Symbiodiniaceae under the dual lifestyle are shown, with a focus on post-WGD ohnolog sequence divergence and differential gene expression.

Our results provide strong evidence that the dual lifestyle has been a key driver of post-WGD genome evolution in the dinoflagellate *D. trenchii*. Our working hypothesis is illustrated in Fig. 4.

Under the null hypothesis of a solely free-living lifestyle, we expect post-WGD adaptations to primarily be driven by fluctuating environmental conditions (e.g., nutrient availability). Under the hypothesis of a dual lifestyle that includes symbiosis, adaptations will also strengthen the maintenance of a stable host-symbiont relationship and efficient nutrient/metabolite exchange within the coral holobiont. Although our results provide stronger support for the free-living phase as the primary driving force behind post-WGD evolution, both lifestyles impact the maintenance and expression divergence of ohnologs. These combined selective forces increase the overall fitness in *D. trenchii*, with the greater expression divergence of ohnologs under elevated temperatures a contributor to the high thermotolerance of this species when it is in symbiosis with corals (*47*). Benefits conferred by WGD to a free-living lifestyle in more variable environments, as well as tailoring of post-WGD duplicates to different lifestyles, primed *D. trenchii* to persist longer in the coral holobiont when faced with thermal stress. Whether symbiosis may also have negative effects on fitness post-WGD is unknown (*48*). It should be noted that the dual lifestyle is widespread in Symbiodiniaceae (*1*), but WGD is not. Although other facultative symbionts within Symbiodiniaceae (e.g., *Cladocopium thermophilum* and *Durusdinium glynnii*) are also known for their thermotolerance (*49*–*51*), WGD was not implicated in these lineages (*8*, *27*). Therefore, the key feature of *D. trenchii* that we are addressing is not dual lifestyle alone, but rather how the capacity for dynamically switching between the symbiotic versus free-living phase affects post-WGD genome evolution and adaptation. Because Symbiodiniaceae propagate to very high densities in coral tissues (10^5^ to 10^6^ cells/cm^2^) (*52*, *53*), the symbiotic phase of *D. trenchii* allows a rapid increase in the population size, particularly of fast-growing genotypes, while resident in host tissues. Consequently, genotypes that have faster growth rates or greater resilience to heat due to WGD-derived adaptations can re-seed free-living populations upon dissociation from the coral due to colony death, bleaching, or other mechanisms of symbiont population control. Repeated cycles of symbiosis followed by the free-living phase may therefore increase the overall fitness of *D. trenchii* populations under the dual lifestyle (*26*). Retention of multiple gene copies combined with fixed, adaptive changes likely makes *D. trenchii* more capable of metabolic maintenance under dynamic, often stressful environments, and hence a more resilient symbiont. Such factors may, in turn, explain the large geographic and expanded host range of *D. trenchii* (*23*) and its well-known capacity for increasing coral survival under heat waves. Therefore, in an intriguing and unexpected twist, WGD, primarily driven by selection under a free-living life phase has converted *D. trenchii* into a coral symbiont able to protect the host coral from thermal stress during symbiosis. *D. trenchii* is also a valuable model for studying the genome-wide impacts of facultative lifestyles.

## MATERIALS AND METHODS

### De novo genome assembly and prediction of protein-coding genes

*D. trenchii* strains CCMP2556 and SCF082 (previously designated UTSD amur-D-MI) originally isolated from an *Orbicella faveolate* and *Acropora muricata* coral colonies, respectively, were each separately cultured and genomic DNA–extracted for genomic sequencing (see Supplementary Text). Chromium libraries were generated for 10x linked-read sequencing and yielded a total of 236.45 giga–base pairs (Gbp) for CCMP2556 and 212.03 Gbp for SCF082. We assessed the ploidy of *D. trenchii* using *k*-mers and GenomeScope2 (*54*), which revealed a distinctive single peak in both isolates indicating a haploid genome as seen in other Symbiodiniaceae (fig. S24).

For each isolate, a preliminary draft genome was assembled de novo using 10x Genomics Supernova v2.1.1. For CCMP2556, the estimated genome coverage (~100×) exceeded the optimal range (38 to 56×) of the Supernova assembler; we subsampled the 1.6 billion reads to 600 million reads (~60× coverage). For SCF082, coverage estimates were observed to be affected due to the presence of contaminant DNA from microbial sources in the sequencing reads; the de novo assembly was generated using all 1.4 billion reads with the flag *–accept_extreme_coverage*.

The presence of putative contaminant scaffolds in the supernova assemblies was investigated using a comprehensive approach adapted from Iha *et al*. (*55*) informed by read coverage, G + C content, taxonomic designation, and de novo transcriptome mapping. Taxon-annotated G + C–coverage plots (fig. S25) were generated using the BlobTools suite v1.1 (*56*) to identify scaffolds in each assembly that deviated by read coverage, taxonomic sequence similarity, and/or G + C content. Read coverage was assessed using BWA v0.7.17, based on mapping of quality-trimmed reads [Longranger v2.2.2 (*57*) ran at default setting] to the genome assembly. The taxonomic identity of scaffolds was assigned on the basis of BLASTN search (*E* ≤ 10^−20^) against genome sequences from bacteria, archaea, viruses, and alveolates in the NCBI nucleotide database (released 10 May 2021). De novo transcriptome assemblies were mapped to the genome assemblies using minimap2 v2.18 (*58*) within which we have modified the codes to account for noncanonical splice sites of dinoflagellates. Scaffolds that were designated as non-dinoflagellate were removed from the assemblies if they lacked mapped transcripts from the corresponding de novo transcriptome assembly, or when <10% of mapped transcripts indicated evidence of introns in the genomes. We considered a scaffold as a putative contaminant if (i) its sequence coverage or G + C content is not within the 1.5 × interquartile range and (ii) it lacks any transcript support defined above. Upon removal of these putative contaminant sequences from the CCMP2556 assembly, the filtered assembly was incorporated in the database as the *D. trenchii* reference for assessing the assembled scaffolds of SCF082 using the same approach.

Publicly available RNA sequencing (RNA-seq) data from previous studies of CCMP2556 (*32*) and SCF082 (*59*) were used to further scaffold the assembled genome sequences (see Supplementary Text). RNA-seq reads for both isolates were first quality-trimmed using fastp (*60*) (mean Phred quality ≥30 across a 4-bp window; minimum read length of 50 bp). For each isolate, the filtered reads were assembled de novo using Trinity v2.11.0 (*61*) independently for each treatment. The transcriptome assemblies for CCMP2556 (791,219 total transcripts) and those for SCF082 (355,411 total transcripts) were mapped to the filtered genome assemblies using minimap2 v2.18 (*58*) that was modified to recognize the noncanonical splice sites of dinoflagellates. The mapped transcripts were then used to scaffold the filtered genome assemblies with L_RNA_Scaffolder (*62*) at default parameters.

A second round of scaffolding was then performed with ARBitR (*63*), which incorporates the distance information from linked-read sequencing data when merging and scaffolding assemblies. Longranger BASIC quality-trimmed linked genome reads (outputs from the standard 10x Genomics data workflow) were mapped to the scaffolded genome assemblies for ARBitR scaffolding, yielding the final genome assemblies: CCMP2556 (assembly size = 1.70 Gb; N50 = 750 kb; 29,137 scaffolds) and SCF082 (assembly size = 1.64 Gb; N50 = 398.5 kb; 44,682 scaffolds) (table S2). The CCMP2556 assembly is the most contiguous reported in Symbiodiniaceae aside from the recent chromosome-level assemblies for *Symbiodinium microadriaticum* (*64*) and *Breviolum minutum* (*65*).

Genome and gene features of dinoflagellates are highly idiosyncratic and atypical of eukaryotes, in part due to noncanonical splice sites (*66*). Therefore, the prediction of protein-coding genes from dinoflagellate genomes requires a comprehensive workflow (https://github.com/TimothyStephens/Dinoflagellate_Annotation_Workflow/; accessed 20 January 2022) tailored for these features, guided by high-confidence evidence (*67*). Here, we adopted a customized workflow integrating the results from multiple methods, guided by available transcript and protein sequences, independently for CCMP2556 and SCF082; see Supplementary Text for details.

### Analysis of whole-genome duplication

We first searched for evidence of collinear gene blocks using MCScanX (*68*) in intraspecies mode (*−b 1*) to identify putative duplicate gene blocks within each genome (i.e., segmental duplication and/or WGD), and in interspecies mode (*−b 2*) to identify syntenic gene blocks between the two genomes. A collinear block is defined as at least five genes conserved in the same orientation and order as a result of segmental duplication and/or WGD events. For each comparison, all-versus-all BLASTP search results were restricted to the top five hits (query or subject coverage >50%; *E* ≤ 10^−5^). Predicted genes from each genome were classified using *duplicate_gene_classifier* (within MCScanX) into singleton, dispersed duplicates (i.e., duplicates separated by >20 genes), proximal duplicates (i.e., duplicates separated by <20 genes), tandem duplicates, and WGD/segmental duplicates (i.e., ohnologs).

Second, we assessed the reconciliation between each gene tree and the species tree; the topological incongruence between the two trees indicates a history of gene duplication or loss (*69*). OrthoFinder v2.3.10 (*70*) was first used to infer homologous gene sets among Suessiales species using BLASTP (*E* ≤ 10^−5^). Multiple sequence alignments were performed with MAFFT v7.487 (*71*) (*-linsi*), from which phylogenetic trees were inferred using FastTree v2.1.11 (*72*) at default parameters. Reconciliation of the gene tree and species tree within OrthoFinder was then used to identify lineage-specific duplication events; those specific to *D. trenchii* indicative of WGD-derived duplicated genes (i.e., ohnologs).

Third, we assessed the impact of WGD on the rate of synonymous substitution (*K*_s_) among all homologous gene sets, using CCMP2556 as the reference, following the wgd pipeline (*73*). Briefly, homologous protein clusters were inferred using a Markov clustering algorithm (*74*) from the previous all-versus-all BLASTP search (used for MCScanX) and aligned using MAFFT (*71*). A phylogenetic tree for each homologous protein cluster was inferred using FastTree2 (*72*) and used to estimate *K*_s_ values for each cluster using codeml implemented in PAML (*75*). A Gaussian mixture model was applied to the *K*_s_ distribution, using a four-component model that provided the best fit for the data according to the Akaike information criterion, yielding a final node-averaged histogram of the *K*_s_ distribution. To estimate the timing of WGD, we first calculated the estimated substitution rate (*r*) per year in Symbiodiniaceae adapting the approach of Ladner *et al*. (*30*) to incorporate genome data and the updated divergence time estimates from LaJeunesse *et al*. (*1*).

We followed Aury *et al*. (*17*) to infer metabolic pathways that were preferentially retained in duplicate following WGD using PRIAM v2 (released January 2018). Briefly, we identified metabolic enzymes that had been uniquely retained as ohnologs or singletons. We then compared the proportion of enzymes uniquely retained as ohnologs to singletons, to the background proportion of the number of ohnologs and singletons annotated in the genome. This tests whether the number of uniquely retained metabolic enzymes for a particular pathway exceeds the background levels that would be expected to occur by random. We additionally required (i) five or more distinct enzymatic proteins to be identified as uniquely retained in either duplicate or singleton and (ii) pathways to be significantly overrepresented in both isolates. The proportion of enzymes coded by genes that were uniquely retained as ohnologs or singletons, compared to their overall proportions in the genome, was used to determine which KEGG pathways (*45*) were preferentially retained in duplicate following WGD.

### Evolution of ohnolog expression

Trimmed RNA-seq reads (above) were mapped to the corresponding genome using HISAT2 v2.2.1 (*--concordant-only*) with a Hierarchical Graph FM index informed by annotated exon and splice sites. Counts of uniquely mapped paired-end reads overlapping with coding sequence regions were then enumerated using *featureCounts --p --countReadPairs --B -C*) implemented in Subread v0.2.3 (*76*). The raw counts were filtered to remove lowly expressed genes using the *filtrByExpr* function in edgeR. Differential gene expression analysis was performed with edgeR using a generalized linear model. We considered genes to be differentially expressed when the false discovery rate < 0.01 and the absolute value of log_2_(fold change) > 1. We compared the difference between lifestyles at two temperatures, i.e., symbiosis versus free-living at 34°C (L-34) and symbiosis versus free-living at 28°C (L-28), and the response to temperature stress in the two lifestyles, i.e., 34°C versus 28°C at free-living (T-Fr) and 34°C versus 28°C in symbiosis (T-Sy).

A WGCNA was performed on all genes in R using the WGCNA package. The variance of normalized counts was calculated using the standard DESeq2 workflow followed by its *varianceStabilizingTransformation*. Because symbiosis is a strong driver of expression in Symbiodiniaceae, using the inferred soft-thresholding power for reducing noise and setting a required threshold for gene correlations would have yielded a mean connectivity of more than 4000 at the inferred power of 6. Therefore, a weighted, unidirectional coexpression network was inferred using a power of 18, the recommended value for signed networks with less than 20 samples. Coexpression modules were inferred using the function *blockwiseModules* that collectively infers signed networks (networkType = “signed,” TOMtype = “signed,” maxBlockSize = 10,000, corType = “bicor,” maxPoutliers = 0.05, pearsonFallback = “individual,” deepSplit = 2, dcuth = 0.999, minModuleSize = 30, reassignThreshold = 0.1, cutHeight = 0.2).

We calculated the adjacencies using a signed network with *bicor* robust correlation coefficient (power = 18, type = “signed,” corFnc = bicor, maxPoutliers = 0.1, pearsonFallback = “individual”). A topological association matrix was then inferred with a signed network and *dissTOM* computed from the product. A hierarchical dendrogram of genes was inferred using *hclust* (method = “average”). The dendrogram was cut using *cutreeDynamic* (deepSplit = 2, minClusterSize = 15, cutHeight = 0.999) and the cut dendrogram merged with *mergeCloseModules* (cutHeight = 0.15, corFnc = bicor, maxPoutliers = 0.1, pearsonFallback = “individual”). Preferential distribution of the different gene duplication categories to WGCNA modules was assessed with a chi-square test and a post hoc analysis performed with the R package *chisq.posthoc.test* (https://github.com/ebbertd/chisq.posthoc.test.git; accessed 25 January 2023).

Expression specificity of ohnologs was assessed using the tau (τ) index (*35*), where τ = 1 indicates highly specific expression, and τ = 0 indicates broad expression. The log-normalized fragments per kilobase million (FPKM) counts were used to calculate τ index scores for those genes with a log_2_(FPKM +1) > 1 in at least one condition following Yanai *et al*. (*35*). The τ indices for the different MCScanX duplication categories were compared using a Kruskal-Wallis rank sum test; pairwise comparisons using Wilcoxon rank sum test with continuity correction and Holm *P* value adjustment were performed to determine differences between the duplication categories. A chi-square test of all significant τ indices (τ ≥ 0.7) was conducted to assess potential biases in expression specificity for treatments among the duplication categories.

### Analysis of posttranscriptional regulation

All-versus-all BLASTN search (query or subject coverage >50%; *E* ≤ 10^−20^) was used to identify shared exonic sequences that have been retained since WGD. For inferring DEU within genes among the treatment conditions, gene models were first broken up into exon “counting bins” using the Python script *dexseq_prepare_annotation.py* from DEXSeq. The relative usage of each exon bin, i.e., the number of transcripts mapping to the bin or to the gene, was then calculated from the HISAT2 BAM file using *dexseq_count.py*. The DEXSeq R package was then used to infer DEU within genes using a generalized linear model, correcting for significance at the gene level using the Benjamini-Hochberg method (*77*).

To examine the conservation of splice junctions in ohnolog pairs, all de novo assembled transcripts were first aligned to the genome using a minimap2 v2.20 (*58*) with code modified to recognize alternative splice sites in dinoflagellates, from which splices sites were identified and annotated using Program to Assemble Spliced Alignments (*78*). Splice sites categorized as alternative acceptor, alternative donor, alternative exon, retained exon, and skipped exon were retained for subsequent analysis. Each identified splice event was assigned two unique identifiers to represent the upstream and downstream positions of the splice event, along with its gene identifier and genomic location. The upstream and downstream 300-bp regions for each splice event were then extracted using the bedtools v2.30 *flank* and *getfasta* functions. An all-versus-all BLASTN search of the extracted splice junction sequences was used to identify sequence similarity (*E* ≤ 10^−5^) between the sequences. Custom Python scripts were used to filter the BLASTN results to identify conserved splice junctions, in which both upstream and downstream regions for a splice event in two ohnologs were significantly similar (*E* ≤ 10^−5^). The splice junction profile for each ohnolog pair was then converted to a binary representation, where the presence of a splice junction in an ohnolog was represented as 1 and the absence of a splice junction represented as 0 (i.e., conserved splice junctions represented as 1 in both ohnologs compared to 0 for those that were not conserved). A Kendall’s rank correlation was then conducted in R to identify ohnolog pairs that exhibited a high level of conservation in splice junctions. An exact binomial test was also performed to identify ohnolog pairs that had diverged in terms of total splice junctions (*P* < 0.05).

## Supplementary Materials

### This PDF file includes:

Supplementary Text Figs. S1 to S25 Legends of tables S1 to S23 References

## References

[1] T. C. LaJeunesse, J. E. Parkinson, P. W. Gabrielson, H. J. Jeong, J. D. Reimer, C. R. Voolstra, S. R. Santos, Systematic revision of Symbiodiniaceae highlights the antiquity and diversity of coral endosymbionts. Curr. Biol. 28, 2570–2580 (2018). 30100341 10.1016/j.cub.2018.07.008

[2] N. Rädecker, C. Pogoreutz, H. M. Gegner, A. Cárdenas, F. Roth, J. Bougoure, P. Guagliardo, C. Wild, M. Pernice, J.-B. Raina, Heat stress destabilizes symbiotic nutrient cycling in corals. Proc. Natl. Acad. Sci. U.S.A. 118, e2022653118 (2021). 33500354 10.1073/pnas.2022653118 PMC7865147

[3] T. Xiang, E. Lehnert, R. E. Jinkerson, S. Clowez, R. G. Kim, J. C. DeNofrio, J. R. Pringle, A. R. Grossman, Symbiont population control by host-symbiont metabolic interaction in Symbiodiniaceae-cnidarian associations. Nat. Commun. 11, 108 (2020). 31913264 10.1038/s41467-019-13963-z PMC6949306

[4] T. C. LaJeunesse, J. Wiedenmann, P. Casado-Amezúa, I. D’ambra, K. E. Turnham, M. R. Nitschke, C. A. Oakley, S. Goffredo, C. A. Spano, V. M. Cubillos, Revival of Philozoon Geddes for host-specialized dinoflagellates, ‘zooxanthellae’, in animals from coastal temperate zones of northern and southern hemispheres. Eur. J. Phycol. 57, 166–180 (2021).

[5] X. Pochon, T. C. LaJeunesse, *Miliolidium* n. gen, a new Symbiodiniacean genus whose members associate with soritid foraminifera or are free-living. J. Eukaryot. Microbiol. 68, e12856 (2021). 10.1111/jeu.12856 33966311

[6] M. R. Nitschke, S. C. Craveiro, C. Brandão, C. Fidalgo, J. Serôdio, A. J. Calado, J. C. Frommlet, Description of *Freudenthalidium* gen. nov. and *Halluxium* gen. nov. to formally recognize clades Fr3 and H as genera in the family Symbiodiniaceae (Dinophyceae). J. Phycol. 56, 923–940 (2020). 32267533 10.1111/jpy.12999

[7] R. A. González-Pech, D. Bhattacharya, M. A. Ragan, C. X. Chan, Genome evolution of coral reef symbionts as intracellular residents. Trends Ecol. Evol. 34, 799–806 (2019). 31084944 10.1016/j.tree.2019.04.010

[8] R. A. González-Pech, T. G. Stephens, Y. Chen, A. R. Mohamed, Y. Cheng, S. Shah, K. E. Dougan, M. D. Fortuin, R. Lagorce, D. W. Burt, Comparison of 15 dinoflagellate genomes reveals extensive sequence and structural divergence in family Symbiodiniaceae and genus *Symbiodinium*. BMC Biol. 19, 73 (2021). 33849527 10.1186/s12915-021-00994-6 PMC8045281

[9] Y. Van de Peer, S. Maere, A. Meyer, The evolutionary significance of ancient genome duplications. Nat. Rev. Genet. 10, 725–732 (2009). 19652647 10.1038/nrg2600

[10] M. E. Schranz, S. Mohammadin, P. P. Edger, Ancient whole genome duplications, novelty and diversification: The WGD radiation lag-time model. Curr. Opin. Plant Biol. 15, 147–153 (2012). 22480429 10.1016/j.pbi.2012.03.011

[11] G. C. Conant, K. H. Wolfe, Turning a hobby into a job: How duplicated genes find new functions. Nat. Rev. Genet. 9, 938–950 (2008). 19015656 10.1038/nrg2482

[12] S. Ohno, U. Wolf, N. B. Atkin, Evolution from fish to mammals by gene duplication. Hereditas 59, 169–187 (1968). 5662632 10.1111/j.1601-5223.1968.tb02169.x

[13] P. P. Singh, J. Arora, H. Isambert, Identification of ohnolog genes originating from whole genome duplication in early vertebrates, based on synteny comparison across multiple genomes. PLoS Comput. Biol. 11, e1004394 (2015). 26181593 10.1371/journal.pcbi.1004394 PMC4504502

[14] M. Kellis, B. W. Birren, E. S. Lander, Proof and evolutionary analysis of ancient genome duplication in the yeast *Saccharomyces cerevisiae*. Nature 428, 617–624 (2004). 15004568 10.1038/nature02424

[15] B. Gallone, J. Steensels, T. Prahl, L. Soriaga, V. Saels, B. Herrera-Malaver, A. Merlevede, M. Roncoroni, K. Voordeckers, L. Miraglia, Domestication and divergence of *Saccharomyces cerevisiae* beer yeasts. Cell 166, 1397–1410.e16 (2016). 27610566 10.1016/j.cell.2016.08.020 PMC5018251

[16] J. Peter, M. De Chiara, A. Friedrich, J.-X. Yue, D. Pflieger, A. Bergström, A. Sigwalt, B. Barre, K. Freel, A. Llored, Genome evolution across 1,011 *Saccharomyces cerevisiae* isolates. Nature 556, 339–344 (2018). 29643504 10.1038/s41586-018-0030-5 PMC6784862

[17] J.-M. Aury, O. Jaillon, L. Duret, B. Noel, C. Jubin, B. M. Porcel, B. Ségurens, V. Daubin, V. Anthouard, N. Aiach, Global trends of whole-genome duplications revealed by the ciliate *Paramecium tetraurelia*. Nature 444, 171–178 (2006). 17086204 10.1038/nature05230

[18] C. L. McGrath, J.-F. Gout, T. G. Doak, A. Yanagi, M. Lynch, Insights into three whole-genome duplications gleaned from the *Paramecium caudatum* genome sequence. Genetics 197, 1417–1428 (2014). 24840360 10.1534/genetics.114.163287 PMC4125410

[19] Y. Maeda, R. Kobayashi, K. Watanabe, T. Yoshino, C. Bowler, M. Matsumoto, T. Tanaka, Chromosome-scale genome assembly of the marine oleaginous diatom *Fistulifera solaris*. Marine Biotechnol. 24, 788–800 (2022). 10.1007/s10126-022-10147-7 35915286

[20] T. Tanaka, Y. Maeda, A. Veluchamy, M. Tanaka, H. Abida, E. Maréchal, C. Bowler, M. Muto, Y. Sunaga, M. Tanaka, Oil accumulation by the oleaginous diatom *Fistulifera solaris* as revealed by the genome and transcriptome. Plant Cell 27, 162–176 (2015). 25634988 10.1105/tpc.114.135194 PMC4330590

[21] D. C. Wham, D. T. Pettay, T. C. LaJeunesse, Microsatellite loci for the host-generalist “zooxanthella” *Symbiodinium trenchi* and other Clade D *Symbiodinium*. Conserv. Gene. Resour. 3, 541–544 (2011).

[22] D. T. Pettay, T. C. Lajeunesse, Microsatellite loci for assessing genetic diversity, dispersal and clonality of coral symbionts in ‘stress-tolerant’clade D *Symbiodinium*. Mol. Ecol. Resour. 9, 1022–1025 (2009). 21564826 10.1111/j.1755-0998.2009.02561.x

[23] D. T. Pettay, D. C. Wham, R. T. Smith, R. Iglesias-Prieto, T. C. LaJeunesse, Microbial invasion of the Caribbean by an Indo-Pacific coral zooxanthella. Proc. Natl. Acad. Sci. U.S.A. 112, 7513–7518 (2015). 26034268 10.1073/pnas.1502283112 PMC4475936

[24] R. Rowan, Thermal adaptation in reef coral symbionts. Nature 430, 742 (2004). 15306800 10.1038/430742a

[25] R. Berkelmans, M. J. H. van Oppen, The role of zooxanthellae in the thermal tolerance of corals: A ‘nugget of hope’ for coral reefs in an era of climate change. Proc. R. Soc. B 273, 2305–2312 (2006). 10.1098/rspb.2006.3567 PMC1636081 16928632

[26] D. Bhattacharya, T. G. Stephens, E. E. Chille, L. F. Benites, C. X. Chan, Facultative lifestyle drives diversity of coral algal symbionts. Trends Ecol. Evol. 39, 239–247 (2024). 37953106 10.1016/j.tree.2023.10.005

[27] K. E. Dougan, R. A. Gonzalez-Pech, T. G. Stephens, S. Shah, Y. Chen, M. A. Ragan, D. Bhattacharya, C. X. Chan, Genome-powered classification of microbial eukaryotes: Focus on coral algal symbionts. Trends Microbiol. 30, 831–840 (2022). 35227551 10.1016/j.tim.2022.02.001

[28] S. Shah, K. E. Dougan, Y. Chen, R. Lo, G. Laird, M. D. A. Fortuin, S. K. Rai, V. Murigneux, A. J. Bellantuono, M. Rodriguez-Lanetty, D. Bhattacharya, C. X. Chan, Massive genome reduction predates the divergence of Symbiodiniaceae dinoflagellates. ISME J. 18, wrae059 (2024). 38655774 10.1093/ismejo/wrae059 PMC11114475

[29] G. P. Tiley, M. S. Barker, J. G. Burleigh, Assessing the performance of Ks plots for detecting ancient whole genome duplications. Genome Biol. Evol. 10, 2882–2898 (2018). 30239709 10.1093/gbe/evy200 PMC6225891

[30] J. T. Ladner, D. J. Barshis, S. R. Palumbi, Protein evolution in two co-occurring types of *Symbiodinium*: An exploration into the genetic basis of thermal tolerance in *Symbiodinium* clade D. BMC Evol. Biol. 12, 217 (2012). 23145489 10.1186/1471-2148-12-217 PMC3740780

[31] H. K. Voris, Maps of Pleistocene sea levels in Southeast Asia: Shorelines, river systems and time durations. J. Biogeogr. 27, 1153–1167 (2000).

[32] A. J. Bellantuono, K. E. Dougan, C. Granados-Cifuentes, M. Rodriguez-Lanetty, Free-living and symbiotic lifestyles of a thermotolerant coral endosymbiont display profoundly distinct transcriptomes under both stable and heat stress conditions. Mol. Ecol. 28, 5265–5281 (2019). 31693775 10.1111/mec.15300

[33] Y. Gabay, J. E. Parkinson, S. P. Wilkinson, V. M. Weis, S. K. Davy, Inter-partner specificity limits the acquisition of thermotolerant symbionts in a model cnidarian-dinoflagellate symbiosis. ISME J. 13, 2489–2499 (2019). 31186513 10.1038/s41396-019-0429-5 PMC6776018

[34] P. Langfelder, S. Horvath, WGCNA: An R package for weighted correlation network analysis. BMC Bioinf. 9, 559 (2008). 10.1186/1471-2105-9-559 PMC2631488 19114008

[35] I. Yanai, H. Benjamin, M. Shmoish, V. Chalifa-Caspi, M. Shklar, R. Ophir, A. Bar-Even, S. Horn-Saban, M. Safran, E. Domany, Genome-wide midrange transcription profiles reveal expression level relationships in human tissue specification. Bioinformatics 21, 650–659 (2005). 15388519 10.1093/bioinformatics/bti042

[36] V. E. Prince, F. B. Pickett, Splitting pairs: The diverging fates of duplicated genes. Nat. Rev. Genet. 3, 827–837 (2002). 12415313 10.1038/nrg928

[37] B. Lehner, Conflict between noise and plasticity in yeast. PLoS Genet. 6, e1001185 (2010). 21079670 10.1371/journal.pgen.1001185 PMC2973811

[38] J. L. Matthews, C. A. Oakley, A. Lutz, K. E. Hillyer, U. Roessner, A. R. Grossman, V. M. Weis, S. K. Davy, Partner switching and metabolic flux in a model cnidarian–dinoflagellate symbiosis. Proc. R. Soc. B 285, 20182336 (2018). 10.1098/rspb.2018.2336 PMC6283946 30487315

[39] I. Yuyama, M. Ishikawa, M. Nozawa, M.-A. Yoshida, K. Ikeo, Transcriptomic changes with increasing algal symbiont reveal the detailed process underlying establishment of coral-algal symbiosis. Sci. Rep. 8, 16802 (2018). 30429501 10.1038/s41598-018-34575-5 PMC6235891

[40] R. N. Silverstein, R. Cunning, A. C. Baker, Tenacious D: *Symbiodinium* in clade D remain in reef corals at both high and low temperature extremes despite impairment. J. Exp. Biol. 220, 1192–1196 (2017). 28108671 10.1242/jeb.148239

[41] D. C. Claar, S. Starko, K. L. Tietjen, H. E. Epstein, R. Cunning, K. M. Cobb, A. C. Baker, R. D. Gates, J. K. Baum, Dynamic symbioses reveal pathways to coral survival through prolonged heatwaves. Nat. Commun. 11, 6097 (2020). 33293528 10.1038/s41467-020-19169-y PMC7723047

[42] D. Abrego, B. L. Willis, M. J. van Oppen, Impact of light and temperature on the uptake of algal symbionts by coral juveniles. PLoS ONE 7, e50311 (2012). 23185603 10.1371/journal.pone.0050311 PMC3504000

[43] M. Herrera, S. G. Klein, S. Campana, J. E. Chen, A. Prasanna, C. M. Duarte, M. Aranda, Temperature transcends partner specificity in the symbiosis establishment of a cnidarian. ISME J. 15, 141–153 (2021). 32934356 10.1038/s41396-020-00768-y PMC7852570

[44] S. B. Matsuda, L. J. Chakravarti, R. Cunning, A. S. Huffmyer, C. E. Nelson, R. D. Gates, M. J. H. van Oppen, Temperature-mediated acquisition of rare heterologous symbionts promotes survival of coral larvae under ocean warming. Glob. Chang. Biol. 28, 2006–2025 (2022). 34957651 10.1111/gcb.16057 PMC9303745

[45] M. Kanehisa, M. Furumichi, Y. Sato, M. Kawashima, M. Ishiguro-Watanabe, KEGG for taxonomy-based analysis of pathways and genomes. Nucleic Acids Res. 51, D587–D592 (2023). 36300620 10.1093/nar/gkac963 PMC9825424

[46] G. C. Conant, K. H. Wolfe, Functional partitioning of yeast co-expression networks after genome duplication. PLoS Biol. 4, e109 (2006). 16555924 10.1371/journal.pbio.0040109 PMC1420641

[47] L. J. Chakravarti, M. J. van Oppen, Experimental evolution in coral photosymbionts as a tool to increase thermal tolerance. Front. Mar. Sci. 5, 227 (2018).

[48] L. Carretero-Paulet, Y. Van de Peer, The evolutionary conundrum of whole-genome duplication. Am. J. Bot. 107, 1101–1105 (2020). 32815563 10.1002/ajb2.1520 PMC7540024

[49] B. C. Hume, C. D'Angelo, E. G. Smith, J. R. Stevens, J. Burt, J. Wiedenmann, *Symbiodinium thermophilum* sp. nov., a thermotolerant symbiotic alga prevalent in corals of the world's hottest sea, the Persian/Arabian Gulf. Sci. Rep. 5, 8562 (2015). 25720577 10.1038/srep08562 PMC4342558

[50] E. J. Howells, A. G. Bauman, G. O. Vaughan, B. C. C. Hume, C. R. Voolstra, J. A. Burt, Corals in the hottest reefs in the world exhibit symbiont fidelity not flexibility. Mol. Ecol. 29, 899–911 (2020). 32017263 10.1111/mec.15372

[51] K. E. Turnham, M. D. Aschaffenburg, D. T. Pettay, D. A. Paz-Garcia, H. Reyes-Bonilla, J. Pinzon, E. Timmins, R. T. Smith, M. P. McGinley, M. E. Warner, T. C. LaJeunesse, High physiological function for corals with thermally tolerant, host-adapted symbionts. Proc. Biol. Sci. 290, 20231021 (2023). 37465983 10.1098/rspb.2023.1021 PMC10354691

[52] E. A. Drew, The biology and physiology of alga-invertebrates symbioses. II. The density of symbiotic algal cells in a number of hermatypic hard corals and alcyonarians from various depths. J. Exp. Mar. Biol. Ecol. 9, 71–75 (1972).

[53] J. W. Porter, L. Muscatine, Z. Dubinsky, P. G. Falkowski, Primary production and photoadaptation in light- and shade-adapted colonies of the symbiotic coral. Proc. R. Soc. B 222, 161–180 (1984).

[54] T. R. Ranallo-Benavidez, K. S. Jaron, M. C. Schatz, GenomeScope 2.0 and Smudgeplot for reference-free profiling of polyploid genomes. Nat. Commun. 11, 1432 (2020). 32188846 10.1038/s41467-020-14998-3 PMC7080791

[55] C. Iha, K. E. Dougan, J. A. Varela, V. Avila, C. J. Jackson, K. A. Bogaert, Y. Chen, L. M. Judd, R. Wick, K. E. Holt, Genomic adaptations to an endolithic lifestyle in the coral-associated alga *Ostreobium*. Curr. Biol. 31, 1393–1402.e5 (2021). 33548192 10.1016/j.cub.2021.01.018

[56] D. R. Laetsch, M. L. Blaxter, BlobTools: Interrogation of genome assemblies. F1000Res 6, 1287–1287 (2017).

[57] P. Marks, S. Garcia, A. M. Barrio, K. Belhocine, J. Bernate, R. Bharadwaj, K. Bjornson, C. Catalanotti, J. Delaney, A. Fehr, Resolving the full spectrum of human genome variation using linked-reads. Genome Res. 29, 635–645 (2019). 30894395 10.1101/gr.234443.118 PMC6442396

[58] H. Li, Minimap2: Pairwise alignment for nucleotide sequences. Bioinformatics 34, 3094–3100 (2018). 29750242 10.1093/bioinformatics/bty191 PMC6137996

[59] E. F. Camp, T. Kahlke, B. Signal, C. A. Oakley, A. Lutz, S. K. Davy, D. J. Suggett, W. P. Leggat, Proteome metabolome and transcriptome data for three Symbiodiniaceae under ambient and heat stress conditions. Sci. Data 9, 153 (2022). 35383179 10.1038/s41597-022-01258-w PMC8983644

[60] S. Chen, Y. Zhou, Y. Chen, J. Gu, fastp: An ultra-fast all-in-one FASTQ preprocessor. Bioinformatics 34, i884–i890 (2018). 30423086 10.1093/bioinformatics/bty560 PMC6129281

[61] B. J. Haas, A. Papanicolaou, M. Yassour, M. Grabherr, P. D. Blood, J. Bowden, M. B. Couger, D. Eccles, B. Li, M. Lieber, M. D. MacManes, M. Ott, J. Orvis, N. Pochet, F. Strozzi, N. Weeks, R. Westerman, T. William, C. N. Dewey, R. Henschel, R. D. LeDuc, N. Friedman, A. Regev, De novo transcript sequence reconstruction from RNA-seq using the Trinity platform for reference generation and analysis. Nat. Protoc. 8, 1494–1512 (2013). 23845962 10.1038/nprot.2013.084 PMC3875132

[62] W. Xue, J.-T. Li, Y.-P. Zhu, G.-Y. Hou, X.-F. Kong, Y.-Y. Kuang, X.-W. Sun, L_RNA_scaffolder: Scaffolding genomes with transcripts. BMC Genomics 14, 604 (2013). 24010822 10.1186/1471-2164-14-604 PMC3846640

[63] M. Hiltunen, M. Ryberg, H. Johannesson, ARBitR: An overlap-aware genome assembly scaffolder for linked reads. Bioinformatics 37, 2203–2205 (2021). 33216122 10.1093/bioinformatics/btaa975 PMC8352505

[64] A. Nand, Y. Zhan, O. R. Salazar, M. Aranda, C. R. Voolstra, J. Dekker, Genetic and spatial organization of the unusual chromosomes of the dinoflagellate *Symbiodinium microadriaticum*. Nat. Genet. 53, 618–629 (2021). 33927399 10.1038/s41588-021-00841-y PMC8110479

[65] G. K. Marinov, A. E. Trevino, T. Xiang, A. Kundaje, A. R. Grossman, W. J. Greenleaf, Transcription-dependent domain-scale three-dimensional genome organization in the dinoflagellate *Breviolum minutum*. Nat. Genet. 53, 613–617 (2021). 33927397 10.1038/s41588-021-00848-5 PMC8110477

[66] J. H. Wisecaver, J. D. Hackett, Dinoflagellate genome evolution. Annu. Rev. Microbiol. 65, 369–387 (2011). 21682644 10.1146/annurev-micro-090110-102841

[67] Y. Chen, R. A. González-Pech, T. G. Stephens, D. Bhattacharya, C. X. Chan, Evidence that inconsistent gene prediction can mislead analysis of dinoflagellate genomes. J. Phycol. 56, 6–10 (2020). 31713873 10.1111/jpy.12947 PMC7065002

[68] Y. Wang, H. Tang, J. D. DeBarry, X. Tan, J. Li, X. Wang, T.-H. Lee, H. Jin, B. Marler, H. Guo, MCScanX: A toolkit for detection and evolutionary analysis of gene synteny and collinearity. Nucleic Acids Res. 40, e49 (2012). 22217600 10.1093/nar/gkr1293 PMC3326336

[69] M. Goodman, J. Czelusniak, G. W. Moore, A. E. Romero-Herrera, G. Matsuda, Fitting the gene lineage into its species lineage, a parsimony strategy illustrated by cladograms constructed from globin sequences. Syst. Biol. 28, 132–163 (1979).

[70] D. M. Emms, S. Kelly, OrthoFinder: Phylogenetic orthology inference for comparative genomics. Genome Biol. 20, 238 (2019). 31727128 10.1186/s13059-019-1832-y PMC6857279

[71] K. Katoh, D. M. Standley, MAFFT multiple sequence alignment software version 7: Improvements in performance and usability. Mol. Biol. Evol. 30, 772–780 (2013). 23329690 10.1093/molbev/mst010 PMC3603318

[72] M. N. Price, P. S. Dehal, A. P. Arkin, FastTree 2–approximately maximum-likelihood trees for large alignments. PLoS ONE 5, e9490 (2010). 20224823 10.1371/journal.pone.0009490 PMC2835736

[73] A. Zwaenepoel, Y. Van de Peer, Wgd—Simple command line tools for the analysis of ancient whole-genome duplications. Bioinformatics 35, 2153–2155 (2019). 30398564 10.1093/bioinformatics/bty915 PMC6581438

[74] A. J. Enright, S. Van Dongen, C. A. Ouzounis, Graph clustering by flow simulation. Nucleic Acids Res. 30, 1575–1584 (2000). 10.1093/nar/30.7.1575 PMC101833 11917018

[75] Z. Yang, PAML 4: Phylogenetic analysis by maximum likelihood. Mol. Biol. Evol. 24, 1586–1591 (2007). 17483113 10.1093/molbev/msm088

[76] Y. Liao, G. K. Smyth, W. Shi, featureCounts: An efficient general purpose program for assigning sequence reads to genomic features. Bioinformatics 30, 923–930 (2014). 24227677 10.1093/bioinformatics/btt656

[77] Y. Benjamini, Y. Hochberg, Controlling the false discovery rate: A practical and powerful approach to multiple testing. J. R. Stat. Soc. Ser. B 57, 289–300 (1995).

[78] B. J. Haas, S. L. Salzberg, W. Zhu, M. Pertea, J. E. Allen, J. Orvis, O. White, C. R. Buell, J. R. Wortman, Automated eukaryotic gene structure annotation using EVidenceModeler and the Program to Assemble Spliced Alignments. Genome Biol. 9, R7 (2008). 18190707 10.1186/gb-2008-9-1-r7 PMC2395244

[79] D. J. Suggett, D. J. Smith, Coral bleaching patterns are the outcome of complex biological and environmental networking. Glob. Chang. Biol. 26, 68–79 (2020). 31618499 10.1111/gcb.14871

[80] T. P. Hughes, J. T. Kerry, S. R. Connolly, J. G. Álvarez-Romero, C. M. Eakin, S. F. Heron, M. A. Gonzalez, J. Moneghetti, Emergent properties in the responses of tropical corals to recurrent climate extremes. Curr. Biol. 31, 5393–5399.e3 (2021). 34739821 10.1016/j.cub.2021.10.046

[81] T. P. Hughes, K. D. Anderson, S. R. Connolly, S. F. Heron, J. T. Kerry, J. M. Lough, A. H. Baird, J. K. Baum, M. L. Berumen, T. C. Bridge, Spatial and temporal patterns of mass bleaching of corals in the Anthropocene. Science 359, 80–83 (2018). 29302011 10.1126/science.aan8048

[82] D. J. Suggett, S. Goyen, C. Evenhuis, M. Szabó, D. T. Pettay, M. E. Warner, P. J. Ralph, Functional diversity of photobiological traits within the genus *Symbiodinium* appears to be governed by the interaction of cell size with cladal designation. New Phytol. 208, 370–381 (2015). 26017701 10.1111/nph.13483

[83] L. Thomas, N. H. Rose, R. A. Bay, E. H. López, M. K. Morikawa, L. Ruiz-Jones, S. R. Palumbi, Mechanisms of thermal tolerance in reef-building corals across a fine-grained environmental mosaic: Lessons from Ofu, American Samoa. Front. Mar. Sci. 4, 434 (2018).

[84] E. F. Camp, J. Edmondson, A. Doheny, J. Rumney, A. J. Grima, A. Huete, D. J. Suggett, Mangrove lagoons of the Great Barrier Reef support coral populations persisting under extreme environmental conditions. Mar. Ecol. Prog. Ser. 625, 1–14 (2019).

[85] P. Buerger, C. Alvarez-Roa, C. W. Coppin, S. L. Pearce, L. J. Chakravarti, J. G. Oakeshott, O. R. Edwards, M. J. H. van Oppen, Heat-evolved microalgal symbionts increase coral bleaching tolerance. Sci. Adv. 6, eaba2498 (2020). 32426508 10.1126/sciadv.aba2498 PMC7220355

[86] L. J. Chakravarti, V. H. Beltran, M. J. H. van Oppen, Rapid thermal adaptation in photosymbionts of reef-building corals. Glob. Chang. Biol. 23, 4675–4688 (2017). 28447372 10.1111/gcb.13702

[87] K. M. Quigley, C. Alvarez-Roa, J.-B. Raina, M. Pernice, M. J. H. van Oppen, Heat-evolved microalgal symbionts increase thermal bleaching tolerance of coral juveniles without a trade-off against growth. Coral Reefs 42, 1227–1232 (2023).

[88] C. R. Voolstra, D. J. Suggett, R. S. Peixoto, J. E. Parkinson, K. M. Quigley, C. B. Silveira, M. Sweet, E. M. Muller, D. J. Barshis, D. G. Bourne, Extending the natural adaptive capacity of coral holobionts. Nat. Rev. Earth Environ. 2, 747–762 (2021).

[89] M. J. Van Oppen, J. K. Oliver, H. M. Putnam, R. D. Gates, Building coral reef resilience through assisted evolution. Proc. Natl. Acad. Sci. U.S.A. 112, 2307–2313 (2015). 25646461 10.1073/pnas.1422301112 PMC4345611

[90] T. C. LaJeunesse, D. C. Wham, D. T. Pettay, J. E. Parkinson, S. Keshavmurthy, C. A. Chen, Ecologically differentiated stress-tolerant endosymbionts in the dinoflagellate genus *Symbiodinium* (Dinophyceae) Clade D are different species. Phycologia 53, 305–319 (2014).

[91] R. Cunning, P. Gillette, T. Capo, K. Galvez, A. C. Baker, Growth tradeoffs associated with thermotolerant symbionts in the coral *Pocillopora damicornis* are lost in warmer oceans. Coral Reefs 34, 155–160 (2015).

[92] R. N. Silverstein, R. Cunning, A. C. Baker, Change in algal symbiont communities after bleaching, not prior heat exposure, increases heat tolerance of reef corals. Glob. Chang. Biol. 21, 236–249 (2015). 25099991 10.1111/gcb.12706

[93] A. F. Powell, J. J. Doyle, Enhanced rhizobial symbiotic capacity in an allopolyploid species of *Glycine* (Leguminosae). Am. J. Bot. 103, 1771–1782 (2016). 27562208 10.3732/ajb.1600060

[94] T. J. Anneberg, K. A. Segraves, Intraspecific polyploidy correlates with colonization by arbuscular mycorrhizal fungi in *Heuchera cylindrica*. Am. J. Bot. 106, 894–900 (2019). 31162645 10.1002/ajb2.1294

[95] M. Te Beest, J. J. Le Roux, D. M. Richardson, A. K. Brysting, J. Suda, M. Kubešová, P. Pyšek, The more the better? The role of polyploidy in facilitating plant invasions. Ann. Bot. 109, 19–45 (2012). 22040744 10.1093/aob/mcr277 PMC3241594

[96] P. Baduel, S. Bray, M. Vallejo-Marin, F. Kolář, L. Yant, The “Polyploid Hop”: Shifting challenges and opportunities over the evolutionary lifespan of genome duplications. Front. Ecol. Evol. 6, 117 (2018).

[97] K. A. Segraves, The effects of genome duplications in a community context. New Phytol. 215, 57–69 (2017). 28418074 10.1111/nph.14564

[98] S. Ohno, *Evolution by Gene Duplication* (Springer-Verlag, 1970).

[99] G. S. Downs, Y.-M. Bi, J. Colasanti, W. Wu, X. Chen, T. Zhu, S. J. Rothstein, L. N. Lukens, A developmental transcriptional network for maize defines coexpression modules. Plant Physiol. 161, 1830–1843 (2013). 23388120 10.1104/pp.112.213231 PMC3613459

[100] M. Sémon, K. H. Wolfe, Preferential subfunctionalization of slow-evolving genes after allopolyploidization in Xenopus laevis. Proc. Natl. Acad. Sci. U.S.A. 105, 8333–8338 (2008). 18541921 10.1073/pnas.0708705105 PMC2448837

[101] S. Xiao, Z. Mou, D. Fan, H. Zhou, M. Zou, Y. Zou, C. Zhou, R. Yang, J. Liu, S. Zhu, Genome of tetraploid fish Schizothorax o'connori provides insights into early re-diploidization and high-altitude adaptation. iScience 23, 101497 (2020). 32905880 10.1016/j.isci.2020.101497 PMC7486454

[102] L. Li, R. Briskine, R. Schaefer, P. S. Schnable, C. L. Myers, L. E. Flagel, N. M. Springer, G. J. Muehlbauer, Co-expression network analysis of duplicate genes in maize (*Zea mays L*.) reveals no subgenome bias. BMC Genomics 17, 875 (2016). 27814670 10.1186/s12864-016-3194-0 PMC5097351

[103] R. De Smet, E. Sabaghian, Z. Li, Y. Saeys, Y. Van de Peer, Coordinated functional divergence of genes after genome duplication in *Arabidopsis thaliana*. Plant Cell 29, 2786–2800 (2017). 29070508 10.1105/tpc.17.00531 PMC5728133

[104] W. Qian, B.-Y. Liao, A. Y.-F. Chang, J. Zhang, Maintenance of duplicate genes and their functional redundancy by reduced expression. Trends Genet. 26, 425–430 (2010). 20708291 10.1016/j.tig.2010.07.002 PMC2942974

[105] C. L. McGrath, M. Lynch, in *Polyploidy and Genome Evolution*, P. Soltis, D. Soltis, Eds. (Springer, 2012), pp. 1–20.

[106] M. Sémon, K. H. Wolfe, Consequences of genome duplication. Curr. Opin. Genet. Dev. 17, 505–512 (2007). 18006297 10.1016/j.gde.2007.09.007

[107] J. Q. Su, X. R. Yang, T. L. Zheng, Y. Tian, N. Z. Jiao, L. Z. Cai, H. S. Hong, Isolation and characterization of a marine algicidal bacterium against the toxic dinoflagellate *Alexandrium tamarense*. Harmful Algae 6, 799–810 (2007).

[108] S. Lin, S. Cheng, B. Song, X. Zhong, X. Lin, W. Li, L. Li, Y. Zhang, H. Zhang, Z. Ji, The *Symbiodinium kawagutii* genome illuminates dinoflagellate gene expression and coral symbiosis. Science 350, 691–694 (2015). 26542574 10.1126/science.aad0408

[109] D. Kim, J. M. Paggi, C. Park, C. Bennett, S. L. Salzberg, Graph-based genome alignment and genotyping with HISAT2 and HISAT-genotype. Nat. Biotechnol. 37, 907–915 (2019). 31375807 10.1038/s41587-019-0201-4 PMC7605509

[110] G. Marçais, C. Kingsford, A fast, lock-free approach for efficient parallel counting of occurrences of k-mers. Bioinformatics 27, 764–770 (2011). 21217122 10.1093/bioinformatics/btr011 PMC3051319

[111] A. L. Delcher, S. L. Salzberg, A. M. Phillippy, Using MUMmer to identify similar regions in large sequence sets. Curr. Protoc. Bioinform., 10–13 (2003). 10.1002/0471250953.bi1003s00 18428693

[112] T. G. Stephens, R. A. González-Pech, Y. Cheng, A. R. Mohamed, D. W. Burt, D. Bhattacharya, M. A. Ragan, C. X. Chan, Genomes of the dinoflagellate *Polarella glacialis* encode tandemly repeated single-exon genes with adaptive functions. BMC Biol. 18, 56 (2020). 32448240 10.1186/s12915-020-00782-8 PMC7245778

[113] W. J. Kent, BLAT—the BLAST-like alignment tool. Genome Res. 12, 656–664 (2002). 11932250 10.1101/gr.229202 PMC187518

[114] B. Haas, A. Papanicolaou, TransDecoder, GitHub (2017); https://github.com/TransDecoder/TransDecoder/.

[115] B. L. Cantarel, I. Korf, S. M. Robb, G. Parra, E. Ross, B. Moore, C. Holt, A. S. Alvarado, M. Yandell, MAKER: An easy-to-use annotation pipeline designed for emerging model organism genomes. Genome Res. 18, 188–196 (2008). 18025269 10.1101/gr.6743907 PMC2134774

[116] V. Ter-Hovhannisyan, A. Lomsadze, Y. O. Chernoff, M. Borodovsky, Gene prediction in novel fungal genomes using an ab initio algorithm with unsupervised training. Genome Res. 18, 1979–1990 (2008). 18757608 10.1101/gr.081612.108 PMC2593577

[117] M. Stanke, O. Keller, I. Gunduz, A. Hayes, S. Waack, B. Morgenstern, AUGUSTUS: Ab initio prediction of alternative transcripts. Nucleic Acids Res. 34, W435–W439 (2006). 16845043 10.1093/nar/gkl200 PMC1538822

[118] I. Korf, Gene finding in novel genomes. BMC Bioinf. 5, 59 (2004). 10.1186/1471-2105-5-59 PMC421630 15144565

[119] L. Fu, B. Niu, Z. Zhu, S. Wu, W. Li, CD-HIT: Accelerated for clustering the next-generation sequencing data. Bioinformatics 28, 3150–3152 (2012). 23060610 10.1093/bioinformatics/bts565 PMC3516142

[120] M. Stanke, O. Schöffmann, B. Morgenstern, S. Waack, Gene prediction in eukaryotes with a generalized hidden Markov model that uses hints from external sources. BMC Bioinf. 7, 62 (2006). 10.1186/1471-2105-7-62 PMC1409804 16469098

[121] F. A. Simão, R. M. Waterhouse, P. Ioannidis, E. V. Kriventseva, E. M. Zdobnov, BUSCO: Assessing genome assembly and annotation completeness with single-copy orthologs. Bioinformatics 31, 3210–3212 (2015). 26059717 10.1093/bioinformatics/btv351

[122] E. Shoguchi, G. Beedessee, K. Hisata, I. Tada, H. Narisoko, N. Satoh, M. Kawachi, C. Shinzato, A new dinoflagellate genome illuminates a conserved gene cluster involved in sunscreen biosynthesis. Genome Biol. Evol. 13, evaa235 (2021). 33146374 10.1093/gbe/evaa235 PMC7875005

[123] S. Capella-Gutiérrez, J. M. Silla-Martínez, T. Gabaldón, trimAl: A tool for automated alignment trimming in large-scale phylogenetic analyses. Bioinformatics 25, 1972–1973 (2009). 19505945 10.1093/bioinformatics/btp348 PMC2712344

[124] D. C. Wham, G. Ning, T. C. LaJeunesse, *Symbiodinium glynnii* sp. nov., a species of stress-tolerant symbiotic dinoflagellates from pocilloporid and montiporid corals in the Pacific Ocean. Phycologia 56, 396–409 (2017).

[125] A. Zwaenepoel, Z. Li, R. Lohaus, Y. Van de Peer, Finding evidence for whole genome duplications: A reappraisal. Mol. Plant 12, 133–136 (2019). 30599206 10.1016/j.molp.2018.12.019

[126] L. Mao, J. L. Van Hemert, S. Dash, J. A. Dickerson, *Arabidopsis* gene co-expression network and its functional modules. BMC Bioinf. 10, 346 (2009). 10.1186/1471-2105-10-346 PMC2772859 19845953

[127] F. Luo, Y. Yang, J. Zhong, H. Gao, L. Khan, D. K. Thompson, J. Zhou, Constructing gene co-expression networks and predicting functions of unknown genes by random matrix theory. BMC Bioinf. 8, 299 (2007). 10.1186/1471-2105-8-299 PMC2212665 17697349

[128] R. D. Cook, Detection of influential observation in linear regression. Dent. Tech. 19, 15–18 (1977).

[129] R. H. Lindeman, P. F. Merenda, R. Z. Gold, *Introduction to Bivariate and Multivariate Analysis* (Scott Foresman and Co., 1980).

[130] U. Grömping, Relative importance for linear regression in R: The package relaimpo. J. Stat. Softw. 17, 1–27 (2007).

[131] Z. Chen, Y. Omori, S. Koren, T. Shirokiya, T. Kuroda, A. Miyamoto, H. Wada, A. Fujiyama, A. Toyoda, S. Zhang, De novo assembly of the goldfish (*Carassius auratus*) genome and the evolution of genes after whole-genome duplication. Sci. Adv. 5, eaav0547 (2019). 31249862 10.1126/sciadv.aav0547 PMC6594761

[132] Y. J. Liew, Y. Li, S. Baumgarten, C. R. Voolstra, M. Aranda, Condition-specific RNA editing in the coral symbiont *Symbiodinium microadriaticum*. PLoS Genet. 13, e1006619 (2017). 28245292 10.1371/journal.pgen.1006619 PMC5357065

